# LSID Tester, a tool for testing Life Science Identifier resolution services

**DOI:** 10.1186/1751-0473-3-2

**Published:** 2008-02-18

**Authors:** Roderic DM Page

**Affiliations:** 1 Division of Environmental and Evolutionary Biology, Institute of Biomedical and Life Sciences, Graham Kerr Building, University of Glasgow, Glasgow G12 8QQ, UK

## Abstract

**Background:**

Life Science Identifiers (LSIDs) are persistent, globally unique identifiers for biological objects. The decentralised nature of LSIDs makes them attractive for identifying distributed resources. Data of interest to biodiversity researchers (including specimen records, images, taxonomic names, and DNA sequences) are distributed over many different providers, and this community has adopted LSIDs as the identifier of choice.

**Results:**

LSID Tester is a web application written in PHP. Given a LSID the application performs seven tests, reporting the results at each step. If all tests are successful the metadata associated with the LSID is displayed, and can be viewed in a range of formats.

**Conclusion:**

The software provides a tool for testing a LSID resolution service.

## Background

A key prerequisite for integrating biological information from diverse sources is the use of globally unique identifiers (GUIDs) to consistently identify objects [1]. One approach to deploying GUIDs is to provide a central authority for assigning and resolving identifiers. This is the strategy adopted by many academic publishers through CrossRef [2], which manages Digital Object Identifiers (DOIs) [3] for journal articles. In some cases a field may be dominated by a single data provider which issues de-facto GUIDs, for example the genomics community uses GenBank accession numbers to identify molecular sequences. However, neither approach works well for the biodiversity community [4], which has large numbers of globally distributed data providers serving diverse kinds of information such as taxonomic names, specimen records, images, and DNA sequences. At the time of writing the Global Biodiversity Information Facility (GBIF) [5] lists some 214 biodiversity data providers, serving a total of 41,139,985 records, mostly of museum specimens. After reviewing various options for GUIDs, the Biodiversity Information Standards (TDWG) organisation [6] has recommended the use of LSIDs.

Life Science Identifiers (LSIDs) were developed to provide globally unique identifiers for objects in biological databases [1]. Although within mainstream bioinformatics relatively few "early adopters" have deployed LSIDs [7], the biodiversity informatics community has adopted LSIDs as its GUID of choice [6]. Among the attractions are the distributed nature of the identifier (no central authority is required for registering or resolving identifiers), the low cost, and the convention that resolving a LSID returns metadata as RDF [8]. The later facilitates integrating information from multiple sources using tools being developed for the Semantic Web [9].

**Figure 1:**
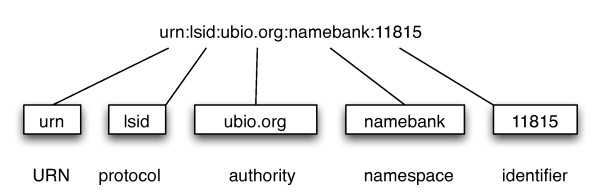
**A Life Science identifier**. A LSID is prefixed with "urn:lsid", then follows the authority, namespace, and identifier components.

**Figure 2:**
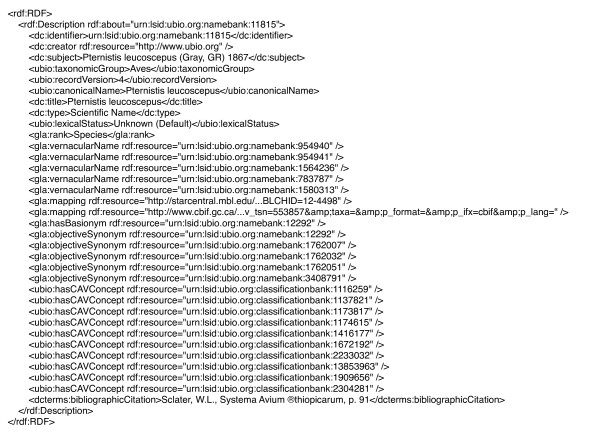
**LSID metadata**. The metadata returned when the LSID urn:lsid:ubio.org:namebank:11815 is resolved. This LSID identifies a record in the uBio database for the taxonomic name *Pternistis leucoscepus*. Note that the RDF shown has been simplified by removing the namespace declarations, and abbreviating some URLs.

Figure 1 shows an example LSID. Each LSID is prefixed by "urn" indicating that the LSID is a Uniform Resource name (URN), "lsid" indicates that the identifier is resolved using the LSID protocol, then follow the authority, namespace, and identifier components. There may also be an optional revision component to indicate the version of the resource. The authority is a domain name that can be resolved by the Internet DNS (typically a domain name owned by the data provider), the namespace and identifier are specific to the data source which provides the resource. In this example the LSID is a taxonomic name in the uBio database [10]. Note that the uniqueness of the LSID is in part guaranteed by the use of Internet domain names, which are globally unique. Providing that the data source ensures that each combination of namespace and identifier is unique within that data source, the LSID itself will be a globally unique identifier. Given a LSID, client software can retrieve metadata and/or data identified by that LSID. Figure 2 shows the metadata corresponding to the LSID urn:lsid:ubio.org:namebank:11815, which identifies a record in the uBio database for the taxonomic name *Pternistis leucoscepus*.

The widely distributed nature of biodiversity data has implications for deploying global identifiers. Providers are unlikely to run a single type of web server, nor are they likely to all use the same web application software. Consequently, there are multiple versions of LSID server software available, including Java, Perl, and .NET implementations [11]. Developers porting servers to new computer programming languages would benefit from having a tool available to test their implementation. Data providers implementing a LSID server would benefit from having a tool to test whether their installation is functioning correctly. The LSID Tester was developed with these two audiences in mind. It is a simple web-based application that tests a LSID service and provides a detailed report on how well the service conforms to the LSID specification [12].

## Implementation

The LSID Tester is written in the PHP programming language, and makes use of the PEAR Net_DNS module [13] written by Eric Kilfoil for LSID resolution discovery. The application caches authority WSDL files and metadata for 24 hours. Metadata is displayed in alternative formats using XSL style sheets, including Oliver Becker's XML to HTML Verbatim Formatter [14]. Graphical displays of metadata use a RDF parser from ARC [15] and require GraphViz [16] to generate the graphs.

## Results and Discussion

### LSID Resolution

A LSID client, such as LSID Tester, resolves a LSID in four steps. Firstly the client discovers the location of the service that can resolve the LSID, for example by querying the DNS service records to find the hostname and TCP/IP service port for the LSID authority. Given the LSID urn:lsid:ubio.org:namebank:11815, querying the DNS for the SRV record for _lsid._tcp.ubio.org returns animalia.ubio.org:80 as the location of the ubio.org LSID service.

Knowing the location of the LSID service, the client appends '/authority/' to the service location, and retrieves the authority WSDL file [17]. This file defines the LSID resolution service, including location and bindings. The LSID standard [12] defines bindings for SOAP, HTTP GET, and FTP. The HTTP GET binding is the mostly widely used, and is the only one the LSID Tester supports at present. For the LSID urn:lsid:ubio.org:namebank:11815 the HTTP GET binding is .

Given the authority WSDL, a LSID client uses its preferred protocol (SOAP, HTTP GET, FTP) to retrieve a second WSDL file (the service WSDL) that specifies how the metadata and/or data corresponding to the LSID can be retrieved. For the LSID urn:lsid:ubio.org:namebank:11815 metadata can be obtained via HTTP GET from .

The client can now retrieve the metadata associated with the LSID by appending ?lsid=urn:lsid:ubio.org:namebank:11815 to this URL.

### Tests

The LSID Tester performs seven main tests:

1. Is the LSID correctly formed?

2. Is the resolution service discoverable?

3. Can it retrieve the authority WSDL?

4. Does the authority WSDL define a HTTP GET binding for the service WSDL?

5. Can it retrieve the service WSDL?

6. Does the service WSDL define a HTTP GET binding for the metadata?

7. Can it retrieve the metadata for the LSID?

**Figure 3:**
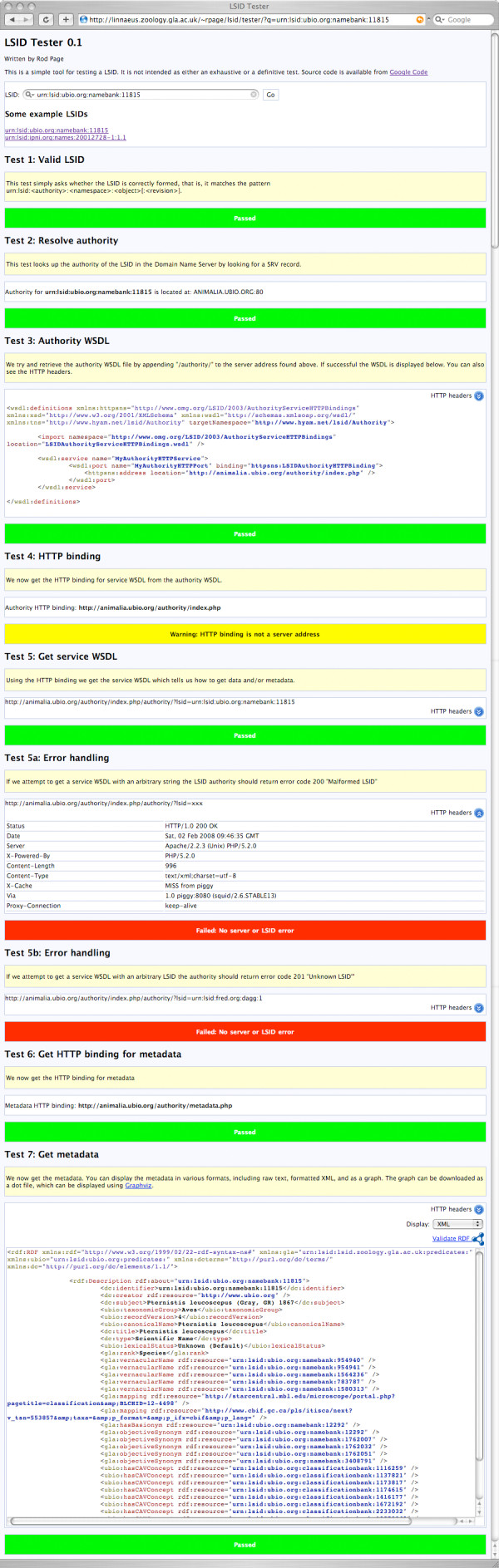
**LSID Tester screenshot**. An instance of LSID Tester resolving the LSID urn:lsid:ubio.org:namebank:11815.

In addition, at step 5 it performs two additional tests. The LSID specification [12] lists error codes a LSID service should use. At test 5 the LSID Tester determines whether the LSID service returns code 200 (MALFORMED_LSID) if supplied with a syntactically invalid LSID, and 201 (UNKNOWN_LSID) if supplied with a syntactically valid LSID with a different authority (i.e., a LSID for which the service is not the authority). At each step (unless a fatal error is encountered) the LSID Tester web page displays the result of the test (Fig. 3). For each HTTP GET call the HTTP headers can be viewed, which can provide useful debugging information (for example, the user can see the HTTP status codes returned by the LSID service). If test 7 succeeds, the metadata associated with the LSID is displayed. Several alternative views are available, such as a formatted XML dump, a raw XML dump, and a graphical display of the metadata. The application also displays a link to the W3C RDF Validation Service [18] so that the user can validate the RDF metadata.

## Conclusion

LSID Tester is a web application for testing LSID resolution services. Given a LSID the application performs seven tests, reporting the results at each step. If all tests are successful the metadata associated with the LSID is displayed, and can be viewed in a range of formats.

## Availability and requirements

**Project Name: **LSID Tester

**Project Home Page: **Source code is available from , and an instance of the application can be viewed at .

**Operating System: **Mac OS X, Linux

**Programming Language: **PHP

**Other Requirements: **Web server, GraphViz

**License: **GNU General Public License version 2

**Any restrictions to use by non-academics: **None

## Competing interests

The author(s) declare that they have no competing interests.
